# Does Electroacupuncture Have Different Effects on Peripheral and Central Sensitization in Humans: A Randomized Controlled Study

**DOI:** 10.3389/fnint.2019.00061

**Published:** 2019-10-15

**Authors:** Zhen Zheng, Linghan Bai, Meredith O’Loughlan, Chun Guang Li, Charlie C. Xue

**Affiliations:** ^1^School of Health and Biomedical Sciences, RMIT University, Melbourne, VIC, Australia; ^2^Spring Health, Ballarat, VIC, Australia; ^3^NICM Health Research Institute, Western Sydney University, Westmead, NSW, Australia

**Keywords:** acupuncture – therapy, central sensitization, peripheral sensitization, hyperalgesia, randomized control experiment

## Abstract

**Background:**

Acupuncture is used to reduce chronic musculoskeletal pain. The common mechanism underlying these types of pain are peripheral and/or central sensitization. In the clinical setting, it is difficult to separate the peripheral from the central component of pain. Heat/capsaicin 45°C/0.075%-induced hyperalgesia provides a stable, human central sensitization model in which the peripheral component is also assessed.

**Aim:**

This randomized, sham-controlled study aimed to investigate the effect of electroacupuncture (EA) on the severity of heat (peripheral sensitization) and mechanical hyperalgesia (central sensitization) in a heat/capsaicin pain model in humans.

**Methods:**

Twenty-six healthy young participants (24 ± 3.9 years) were recruited. After baseline assessment, heat/capsaicin 45°C/0.075% was applied to the non-dominant forearm to induce hyperalgesia. The primary outcome measures were the size of the area of mechanical hyperalgesia, intensity of pain to heat stimulation and heat pain thresholds. The intensity of pain was recorded using modified 10-cm visual analogues scales (VAS). Participants were assessed at 70 min after the initial application of capsaicin then randomly allocated to receive either real electroacupuncture (REA, *n* = 14) or sham non-invasive EA (SEA, *n* = 12) for 30 min. The main outcome measures were assessed again immediately and then 90 min following EA. Credibility of blinding was assessed. Data were analyzed with *t*-tests or analysis of variance (ANOVA) where appropriate.

**Results:**

After the model was established, the area of mechanical hyperalgesia was formed (55.64 cm^2^), as was heat hyperalgesia, as the rating to heat stimulation, increased from 2/10 to 6/10. The REA and SEA groups were comparable. Immediately after the allocated acupuncture treatment, the rating to heat stimulation was statistically significantly lower in the REA group (2.94 ± 1.64) than in the SEA group (4.62 ± 2.26) (*p* < 0.05). The area of mechanical hyperalgesia reduced significantly without any group difference. No group difference was detected in heat pain threshold. Blinding of the participants was successful.

**Conclusion:**

Peripheral and central sensitization in the heat/capsaicin 45°C/0.075% model responded to EA differently, suggesting that acupuncture analgesia could vary, depending on the types of pain. This observation may explain some inconsistent findings from clinical trials of acupuncture.

## Introduction

Acupuncture, an ancient treatment, has been used in China for more than 2000 years. Nowadays this therapy is practiced around the world, in particular for pain relief. Systematic reviews of acupuncture for pain often show a high heterogeneity among included studies (Deare et al., 2013; Chau et al., 2018). Some may be due to the variations in treatment protocols (Jan et al., 2017), the skills of practitioners (Hao et al., 2013), and others may be due to the diverse types of pain. For instance, chronic non-specific low back pain is understood to be a heterogenous group and sub-types exists (Huijnen et al., 2015). To date, however, it is unclear which type of pain responds better to acupuncture treatment. According to the neural mechanism, peripheral, and central sensitization are the two key mechanisms underlying many forms of pain (Treede et al., 1992). The two are likely to co-exist in all pain, but chronic pain tends to have more input from the central nervous system as the pain or hyperalgesia is often beyond the initial injury site (Woolf, 2011).

In a clinical pain state, it is difficult to isolate central from peripheral inputs. There are, however, a few reliable human models for hyperalgesia, such as those induced by topical application of capsaicin (a chili pepper) (Zheng et al., 2000), a burn injury to the skin (Pedersen et al., 1996), or a combination of capsaicin and heat (Petersen and Rowbotham, 1999). Those models have been used to study the potential mechanisms of various pain medications (Petersen-Felix et al., 1995; Petersen et al., 1997, 2001, 2003), and found to be very reliable and useful. Hyperalgesia is an increased response to painful or nearly painful stimulation, which could be heat, or mechanical, and can be easily measured in humans. In a capsaicin model, heat hyperalgesia, that is the enhanced sensitivity to heat stimulation at the site of application of capsaicin, has been found to be due to peripheral sensitization; whereas mechanical hyperalgesia, enhanced sensitivity to mechanical stimulation far beyond the area of capsaicin, is due to central sensitization (LaMotte et al., 1991; Koltzenburg et al., 1992). This model provides an excellent opportunity to study the effect of acupuncture on peripheral and central sensitization in pain.

Mechanical hyperalgesia in a capsaicin model tends to reduce over a short time, making it difficult to study the effect of intervention. The heat/capsaicin 45°C/0.075% model combines two types of nociceptive stimulation methods (topical capsaicin and heat) to produce a stable and long-lasting hyperalgesia model when compared to using either method alone (Petersen and Rowbotham, 1999; Dirks et al., 2003). The mechanical hyperalgesia state can be maintained by periodically repeated application of heat stimulation (Dirks et al., 2000; Petersen et al., 2003).

### Aim

The aims of this sham-acupuncture controlled study were to:

1. assess the effect of electroacupuncture (EA) on the magnitude of mechanical hyperalgesia in a heat/capsaicin 45°C/0.075% pain model, which represents central sensitization;
2. compare the effect of EA on the magnitude of heat pain threshold (HPT) and rating to heat stimulation in the same heat/capsaicin 45°C/0.075% model, which represents peripheral sensitization.

We hypothesized that both heat and mechanical hyperalgesia would be reduced by REA when compared with SEA.

## Materials and Methods

This one-session, participant-assessor blinded, randomized, sham acupuncture controlled study was approved by the Human Research Ethic Committee of RMIT University (Reference No. 13/07). The study was conducted at the clinical research lab of RMIT Chinese Medicine Research Group, Bundoora West Campus. The laboratory was a quiet, well-lit and temperature controlled (20–22°C) room.

### Recruitment and Selection of Participants

Volunteers were recruited from the local community via advertisements posted on the RMIT Bundoora West campus; and were screened according to the following inclusion and exclusion criteria. To be included, participants must have been (1) Aged between 18 and 40 years old healthy volunteers; (2) free from acute or chronic pain; (3) agree to make themselves available for the period of the study; (4) provide a written consent for participation. Volunteers who met one of the following criteria were excluded: (1) using simple analgesics, anti-inflammatory agents, anti-anxiety agents, anti-depressants or anti-psychotic agents; (2) having or having had one or more of the following conditions: stroke, epilepsy, diabetes, severe alcoholism, peripheral vascular disease, peripheral neuropathy, psychosis, heart disease, impaired circulation in hands or feet; or (3) wearing a cardiac pacemaker, having metal implant, being allergies to chili pepper or adhesive paper or being pregnant. To ensure the success of blinding, volunteers who had acupuncture treatment in the past 1 year were excluded as they might be able to differentiate sham acupuncture from real acupuncture.

Participants were given a Plain Language Statement and a verbal explanation regarding any questions about the experiment, and were notified that they were free to withdraw at any time. A signed consent form was obtained from each participant prior to the commencement of the experiments.

### Heat/Capsaicin 45°C/0.075% Pain Model

To produce the heat/capsaicin 45°C/0.075% model, moderate thermal stimulation (45°C) was given for 5 min in the middle of the non-dominant forearm. Capsaicin cream (Zotrix HP cream, 0.075% capsaicin) was then applied topically to the heated area for 30 min to achieve further sensitization. The sensitized area was rekindled at 40°C for 5 min periodically throughout the session to maintain the status of mechanical hyperalgesia. Previous studies proved this being a reliable method to provide mechanical hyperalgesia (Petersen and Rowbotham, 1999; Dirks et al., 2003). The thermal stimulation was delivered via Advanced Thermosensory Stimulator (Medoc TSA 2001, Medoc, Ramat Yishai, Israel), which has a computer controlled thermode with a surface area of 3 cm × 3 cm.

### Outcome Measurements

#### Area of Mechanical Hyperalgesia

**FIGURE 1:**
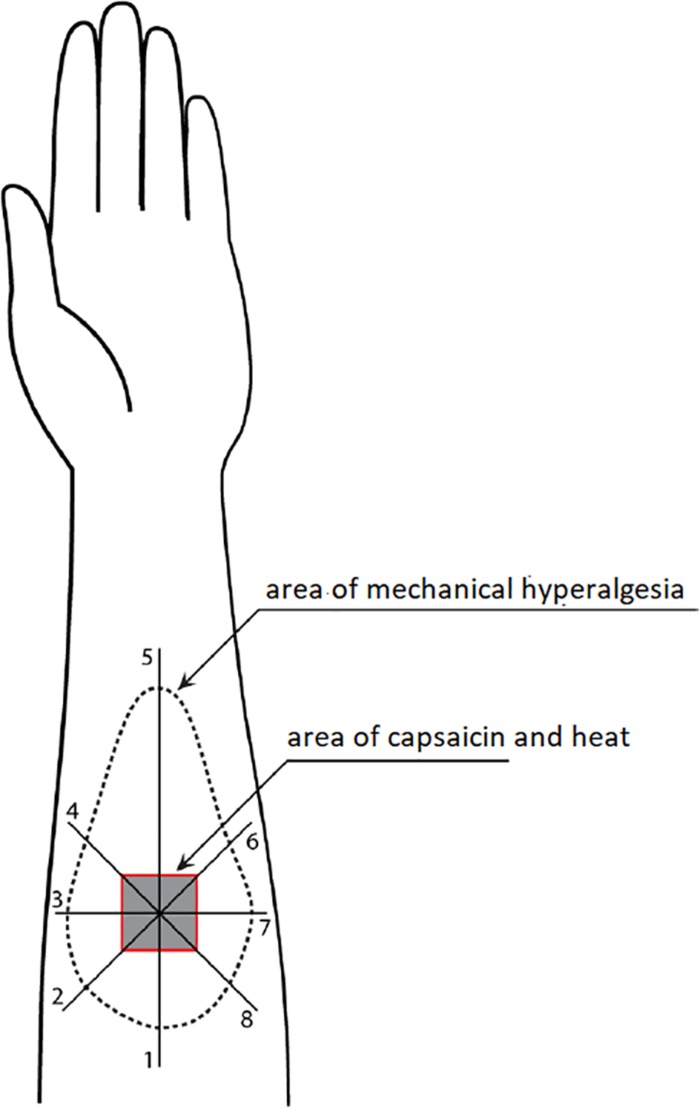
Illustration of measurement for the area of mechanical hyperalgesia.

Figure 1 illustrates how the area of mechanical hyperalgesia was measured. A von Frey filament (4.93) was applied at a point 8 cm away from the center of the capsaicin area and then moving toward the center at approximately 1 cm intervals every 2 s. Participants were provided with written instruction about skin hypersensitivity and asked to report when the filament caused a definite increase in the magnitude of pricking sensation or pain. This point was marked on the skin using a colored fiber-tipped pen and this process was repeated in a pattern of eight radial lines from the center of the capsaicin site (Zheng et al., 2000, 2009). The resulting eight points were connected to define the outline of mechanical hyperalgesia, which was then transferred onto a transparent plastic sheet. The size of the area was then measured with a Digital Planimeter (Planix, Tamaya & Company Ltd., Japan).

#### Heat Pain Threshold

Heat pain threshold is the lowest temperature that the participants perceived as painful. It was measured on the forearm of both capsaicin treated and non-capsaicin sides using a 3 cm × 3 cm thermode (Medoc TSA 2001). The test used was a modified Limits test with four continuous heat stimulations. The baseline temperature of the thermode was 32°C. After the test began, it increased at a rate of 1°C/s. Participants indicated when the pain threshold (the point they began to feel the stimulation became painful) was reached by pressing the Yes mouse button. The temperature was automatically recorded by the computer and the thermode temperature immediately returned to baseline. The cut-off temperature was set at 50°C. The next stimulation was given 4–6 s after the restoration of the baseline. The HPT was calculated as the average of four measurements.

#### Intensity of Pain to Long Thermal Stimulation

Painfulness to the long thermal stimulation (40°C, 1 min) was measured during baseline measurement and in the first minute during each rekindling, using a modified VAS with 0 being no sensation, 5 being just painful and 10 being worst pain possible (Zheng et al., 2014). Participants were instructed to rate their sensations at 5, 10, 15, 25, 35, 45, and 55 s to the stimulation.

### Method of Randomization and Participant-Assessor Blinding

Before the acupuncture intervention, participants drew a sealed envelope which contained a random number, indicating the assignment to either REA or SEA group. The random number sequence was generated with Microsoft Excel (Microsoft Office, Windows version) by an independent investigator who was involved in neither acupuncture intervention nor testing. The acupuncturist was the only person who knew the group assignment, and was blinded from the outcome measures. The investigator who performed the outcome measures was blinded from the group assignment and the process of the acupuncture intervention. During the acupuncture treatment, participants lay on a treatment bed in supine posture and their vision to the site of the acupuncture was blocked. By the end of the treatment they were informed not to disclose any information about the nature of their treatment to the investigator who performed tests on them. During the data analysis stage, an independent investigator conducted the data analysis.

### Acupuncture Interventions

#### Selection of Acupoints

Four acupoints on both sides were selected: Zusanli (ST36) and Fenglong (ST40), Hegu (LI4), and Shousanli (LI10), based on our previous studies on clinical (Zheng et al., 2008) and experimental pains (Zheng et al., 2010). These points were commonly used for pain treatment. Acupuncture points were located according to the textbook description (Cheng et al., 1999) by a registered acupuncturist.

#### REA

Needles were inserted into acupoints to a depth of 15–25 mm, and were manipulated to achieve De Qi sensations (described as soreness, numbness, or distension at the needling site). A bipolar electrical acupuncture stimulator (MEE 501, Australia) was connected to the eight acupoints on both sides of the body using four pairs of electrodes. The parameter for REA was dense-disperse (D-D) mode with alternating frequency between 5 and 15 Hz. The intensity was adjusted to a strong but comfortable lever with visible muscle contraction; and was adjusted twice during the treatment to compensate the participants’ tolerance. The duration of REA treatment was 25 min.

#### SEA

**FIGURE 2:**
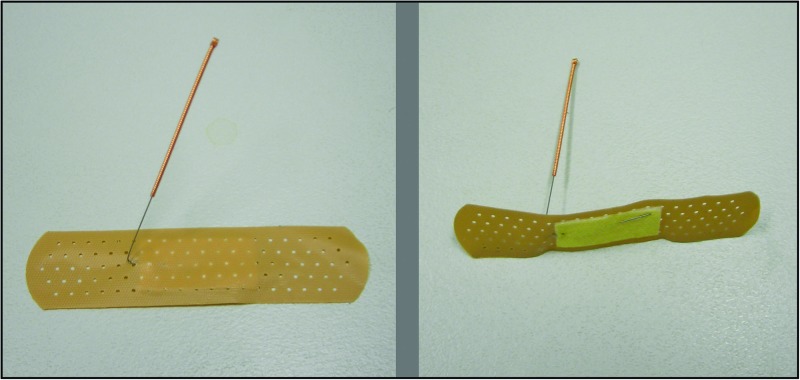
Photos of sham acupuncture.

A non-invasive sham that was tested and used in a previous study was adopted in this trial (Zheng et al., 2010; Figure 2). First, an empty plastic guide tube was tapped onto the non-acupoint, that are not along any meridians but relatively close to the real point, to produce the discernible sensation; then bent needles with adhesive bandage were taped to the dermal surface of each acupoint; and was connected to a mock electrical acupuncture stimulator without delivering electrical stimulation. The stimulator was placed within the participant’s sight, showing a continuously flashing light. During the treatment the acupuncturist adjusted the stimulator twice, and pressing the bended needles to the skin three time to produce some discernible sensation. De Qi sensation was not intended to be produced during SEA. For both REA and SEA, acupuncture needles were eight 0.3 × 40 mm sterile single-use needles with guide tube (Huato, Suzhou Medical Appliance Company, China).

### Statistical Analysis and Sample Size

Data were summarized as means and standard deviations (SD) in the tables, and means and standard error of mean (SEM) or percentage in the figures. Two-way ANOVA (group and time) with one repeated measure (time) was used to analyze the effect of intervention on the size of mechanical hyperalgesia area, heat pain threshold and VAS rating to long thermal stimulation with the rating to the first 5 s stimulation was used for analysis. ANCOVA (Analysis of covariance) was used to adjust for age to verify the results as age was not comparable between the groups. A *p*-value of 0.05 was considered statistically significant. For parametric data, independent-*t*-tests were used to assess the comparability of REA and SEA on age, pain thresholds, pain ratings and areas of mechanical hyperalgesia. For the categorical data such as gender, hand dominance, sham procedure credibility and acupuncture perception, the chi-squared test was used.

Data analysis was conducted by an independent researcher who was blinded to the group assignment and tests. All of the statistical analyses were conducted using the Statistical Package for the Social Sciences (SPSS, Windows Version 25.0).

This was the first study assessing the anti-hyperalgesia effect in human participants, no previous data was available for the sample size calculation. A previous study showed 12–14 participants in each group were sufficient for psychophysics studies in healthy humans of this nature (Petersen et al., 2001; Zheng et al., 2010). At the end of the study, *post hoc* sample size calculations were conducted.

### Procedure

**FIGURE 3:**
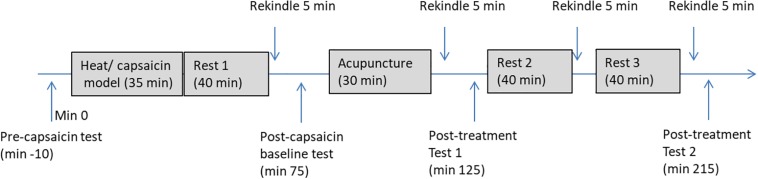
A flow chart of the experiment procedure. Pre-capsaicin test includes HPT, rating to long-thermal stimulation; Post-capsaicin test, post-treatment test 1, and post-treatment test 2 includes HPT, rating to long-thermal stimulation and area of mechanical hyperalgesia.

Figure 3 illustrates the detailed procedure used in the experiment. At the start of the experiment, participants were familiarized with the HPT test and the use of VAS. During the baseline (pre-capsaicin) measurement, HPT in both forearm and rating to the long thermal stimulation (40°C for 1 min) were measured. After this, hyperalgesia was produced on the non-dominant arm of the participant by heating (45**°**C) an area of 3 cm × 3 cm in the middle of the forearm for 5 min followed with application of a thick layer of capsaicin cream (0.075%) in the sensitized site for 30 min. Participants were allowed to rest for 35 min after the removal of the capsaicin cream. The sensitized area was then rekindled with heat stimulation at 40**°**C for 5 min and subjects was asked to rate the stimulation every 10 s during the first minute. Area of the mechanical hyperalgesia was measured immediately after the rekindling and the previous measurements at baseline (pre-capsaicin) were repeated. Participants were then randomly allocated to receive a 25-min intervention of either EA or SEA delivered by an acupuncturist who was blinded to the group allocation. Forty minutes after the first rekindling, the second rekindling was given and the same measurements were taken. The third rekindling was given 40 min after the second rekindling, and no measurements were taken after this rekindling. The last rekindling was given 40 min after the third one, and the previous measurements (area of hyperalgesia, HPT, pain to heat stimulation) were repeated. The whole session took about four and half hours.

## Results

### Demographic and Baseline Data

A total 26 participants were recruited within 3 months, according to the inclusion and exclusion criteria. All 26 participants completed the experiment, and no serious adverse effects were reported. Demographic and baseline data are presented in Table 1. Fifteen of the 26 participants were male, and 11 were female. The average age was 24.90 ± 4.12 (*Mean* ± *SD*), ranging from 18 to 32. All participants developed mechanical and heat hyperalgesia, after the heat/capsaicin 45°C/0.075% applications. Ratings to heat stimulation in the capsaicin area were increased from 2/10 to 6/10 in both groups, i.e., from not-painful to be painful; and the area of mechanical hyperalgesia was detectable and was on average over 50 cm^2^. The EA and SEA groups were comparable on demographic data as well as baseline hyperalgesia data, except for the REA group being younger than the SEA group.

**TABLE 1:** Demographic data and baseline data of heat and mechanism hyperalgesia in REA and SEA groups (Mean ± SD).

**Stage**		**REA (*n* = 14)**	**SEA (*n* = 12)**	**Statistical tests**	***P*-value**
Demographic data	Age (Mean ± SD)	22.64 ± 3.92	25.83 ± 3.49	*t* = 2.18	0.039^∗^
	Gender (Male: Female)	9: 5	6: 6	χ^2^(df = 1) = 0.462	0.692
	Dominant hand (Right: Left)	14: 0	11: 1	χ^2^(df = 1) = 0.271	0.462
Pre-capsaicin	HPT: non-capsaicin site (°C)	44.04 ± 3.08	43.55 ± 3.11	*t* = −0.405	0.689
	HPT capsaicin site (°C)	42.59 ± 3.24	41.95 ± 3.04	*t* = −0.513	0.613
	Area of mechanical hyperalgesia (cm^2^)	N/A	N/A		
	VAS rating first 5 s (out of 10)	2.33 ± 2.00	2.03 ± 1.87	*t* = 0.872	0.695
Post-capsaicinBaseline data	HPT: non-capsaicin site (°C)	43.46 ± 2.63	42.75 ± 2.85	*t* = −0.661	0.513
	HPT capsaicin site (°C)	42.54 ± 1.87	41.47 ± 1.80	*t* = −*1.491*	0.149
	Area of mechanical hyperalgesia (cm^2^)	54.99 ± 16.58	56.28 ± 19.97	*t* = *0.181*	0.858
	VAS rating first 5 s (out of 10)	6.47 ± 1.72	6.75 ± 1.55	*t* = *0.545*	0.631

*HPT, heat pain threshold; VAS, Visual analogue scale; REA, real electrical acupuncture; SEA, sham electroacupuncture; SD, standard deviation; ^∗^statistically significant difference.*

After the application of capsaicin, HPT was also slightly reduced on the capsaicin treated site in both REA and SEA group, but the changes were not statistically significant (SEA group *t* = 0.553, *p* = 0.593; REA group *t* = 0.451, *p* = 0.663). Similar changes were on the non-capsaicin treated side.

### The Effect of REA on the Area of Mechanical Hyperalgesia

**FIGURE 4:**
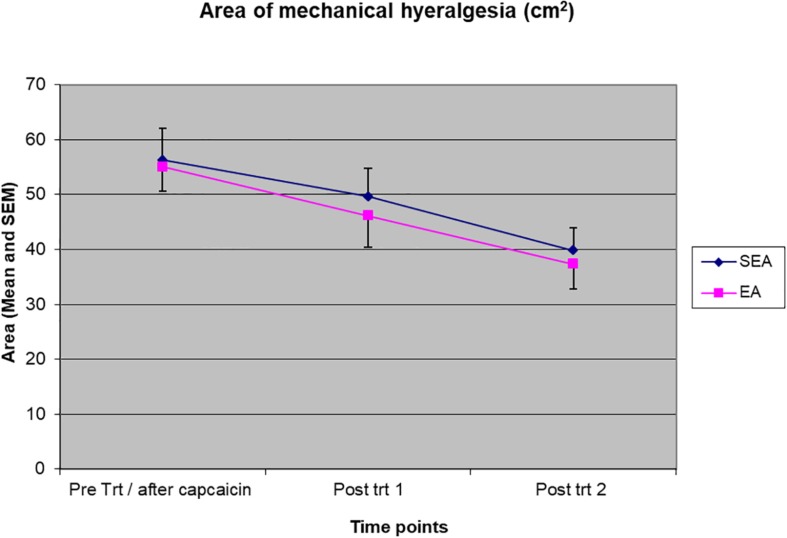
The magnitude of the area of mechanical hyperalgesia before intervention/after capsaicin (Pre trt/after capsaicin), immediately after (Post trt 1) and 90 min after (Post trt 2) intervention in REA and SEA groups. Data are expressed as mean ± SEM.

The area of mechanical hyperalgesia was measured after REA or SEA and during the follow up. Two-way ANOVA showed a statistical significant time effect (*F*_(__2__,__48__)_ = 12.134, *p* < 0.001) but no treatment group by time interaction (*F*_(__2__,__48__)_ = 0.053, *p* = 0.948), indicating the area of mechanical hyperalgesia was reduced in a similar manner in both REA and SEA groups (Figure 4). When ANCOVA was used to adjust for age, the results did not change significantly (group by time: *F*_(__2__,__46__)_ = 0.145, *p* = 0.865).

### The Effect of REA on the Pain Rating to Long-Thermal Stimulation

**FIGURE 5:**
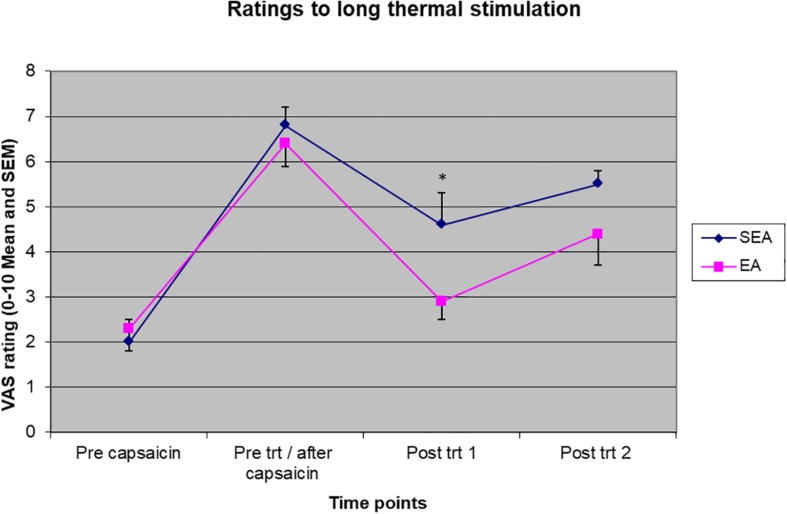
Visual analogues scales pain rating to long thermal stimulation before capsaicin, before intervention/after capsaicin (Pre trt/after capsaicin), immediately after (Post trt 1), and 90 min after (Post trt 2) intervention in REA and SEA groups. Data are expressed as mean ± SEM. ^∗^indicates statistically significantly difference between EA and SEA, *p* < 0.05.

Two-way ANOVA showed there were a statistically significant time effect (*F*_(__3__,__72__)_ = 57.566, *p* < 0.001) and treatment group by time interaction (*F*_(__3__,__72__)_ = 3.173, *p* = 0.029) to pain rating to long-thermal stimulation. As shown in Figure 5, ratings to heat stimulation increased in both groups after heat/capsaicin 45°C/0.075% model, indicating the development of heat hyperalgesia. After the acupuncture treatment, the REA group rated the pain far less than SEA group did. *Post hoc* analysis showed a statistically significant difference at the time point immediately after treatment (REA: 2.94 ± 1.64, SEA: 4.62 ± 2.26, *t* = 2.14, *p* = 0.045). There was no difference in other time points. When ANCOVA was used to adjust for age, the group by time interaction had a trend to be statistically significantly different (*F*_(__3__,__69__)_ = 2.718, *p* = 0.051).

### The Effect of EA on HPT

**FIGURE 6:**
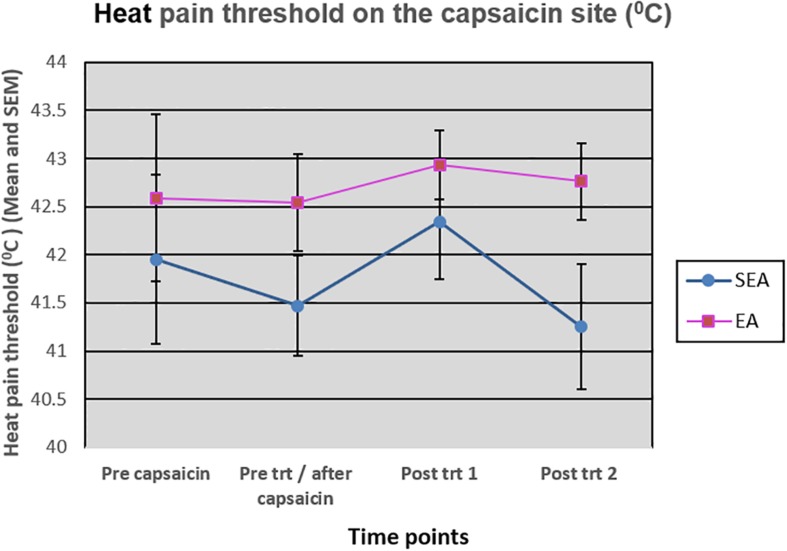
Heat pain threshold on the capsaicin site before capsaicin, before intervention/after capsaicin (Pre trt/after capsaicin), immediately after (Post trt 1), and 90 min after (Post trt 2) intervention in REA and SEA groups. Data are expressed as mean ± SEM.

On the capsaicin-treated site, HPT was reduced slightly after the application of heat/capsaicin 45°C/0.075%, and increased slightly after EA (rekindling 2). After rekindling 4, there was a reduction in the HPT in SEA group, while the HPT in REA group remained relatively unchanged. A two-way ANOVA showed that there was neither statistically significant time effect (*F*_(__3__,__72__)_ = 1.586, *p* = 0.200) nor group by time interaction (*F*_(__3,72__)_ = 0.866, *p* = 0.463) (Figure 6). Similar results were found on the HPT measured on the capsaicin not-treated side.

### Credibility of Sham Acupuncture

At the end of the experiment, a valid questionnaire was used to assess the credibility of sham acupuncture that was used by authors previously (Zheng et al., 2008). Twenty-four participants completed this questionnaire. There was no difference between the REA and SEA groups on guessing which group they belonged to, indicating the blinding procedure was successful (Table 2). The reasons given for the choice were comparable between the groups (Table 2).

**TABLE 2:** Participants’ perception of treatment and reasons for the choice.

**Subject’s answer**	**Frequency of answer in each group (number)**		**Statistical test**	
		
	**REA (*n* = 14)**	**SEA (*n* = 12)**	**χ^2^ (df)**	***P*-value**
**Participants’ perception of treatment**				
Which group were you in?				
I had Real acupuncture	10	6	1.388 (3)	0.708
I had placebo/sham acupuncture	1	2		
Don’t know	2	3		
Missing	1	1		
Reasons for the choice				
Manner, attitude, or words of acupuncturist	1	1	2.019 (4)	0.732
Sensation of acupuncture stimulation	7	4		
Result of the treatment	0	1		
The acupuncture procedure	2	3		
Missing	4	3		
**Participant’s rating to the sensation of acupuncture treatment**				
No pain	1	6	6.378 (3)	0.095
Slight/mild pain	9	4		
Moderate pain	3	1		
Missing	1	1		

*REA, real electrical acupuncture; SEA, sham electroacupuncture.*

### Rating to Acupuncture Stimulation and Side Effects

In the above-mentioned questionnaire, participants were asked to rate the sensation they felt when given acupuncture stimulation (Table 2). More people in the REA group rated the treatment being mild to moderate pain (12/13) when compared with the SEA group (5/11). However, there was no difference between the two groups.

None of the participants reported any side effects such as nausea or dizziness during or after the experiment.

## Discussion

In this experiment, capsaicin combined with repeated heat stimulation successfully produced mechanical and heat hyperalgesia state in both REA and SEA groups. The area of mechanical hyperalgesia, HPT, and VAS rating before acupuncture were comparable between the two groups. REA and SEA group did not differ on the reduction of the area of mechanical hyperalgesia, however, REA was more effective in reducing heat hyperalgesia as indicated with rating to heat stimulation when compared with SEA, but not on the changes in HPT. Peripheral and central sensitization in the heat/capsaicin 45°C/0.075% model responded to REA differently, suggesting acupuncture analgesia could vary depending on the types of pain.

### Strengths of the Study

Firstly, this psychophysics study followed the design of a randomized-controlled trial (RCT) to enhance the internal validity of the results. A few strategies were used to minimize allocation and performance bias. Participants were randomized on the day of the experiment and only one person was aware of the randomization code. To control the placebo effect and to successfully blind the participants, acupuncture naïve and healthy participants were recruited. A pre-tested, valid sham acupuncture control (Lao et al., 2004; Zheng et al., 2010) was used to blind participants. The success of blinding was also assessed with a valid acupuncture credibility questionnaire (Zheng et al., 2008). The study acupuncturist could not be blinded, however, the assessor was blinded from the group allocation. Secondly, both mechanical and heat hyperalgesia were assessed with well-accepted methods that have been used in other hyperalgesia studies and by the authors (Park et al., 1995; Zheng et al., 2000, 2009) and in studies examining the effects of analgesics (Petersen-Felix et al., 1995; Petersen et al., 1997, 2001, 2003; Burns et al., 2006). Thirdly, confounding factors were considered. Before the experiment, participants were given training sessions to be familiar with the experimental procedure. The study was conducted in a temperature-controlled room (20–22°C) to minimize the impact of room temperature on pain perception (Pertovaara et al., 1996). All of those strategies have enhanced the reliability and validly of the study results.

### Limitations

The major limitations of this study are the relatively small sample size, a lack of a no-treatment group and the form of EA. Based on previous similar psychophysics studies, we considered 12–14 participants in each group would be sufficient to provide an estimate of the anti-hyperalgesic effect of acupuncture. Using the data obtained immediately after acupuncture, we calculated the sample size needed to detect the group differences with 80% power in the area of mechanical hyperalgesia (*n* = 972 in total), VAS rating to the long thermal stimulation (*n* = 50 in total), and heat pain threshold (*n* = 204 in total). The small number of participants may have impacted on the group difference in heat hyperalgesia, as the study had only half of the required sample of 50 participants, indicated by our *post hoc* sample size calculation. However, this should not have impacted on the outcome of mechanical hyperalgesia.

Other researchers have shown that hyperalgesia is stable in the heat/capsaicin 45°C/0.075% model, and we also confirmed this in our pilot tests prior to the current study (data not presented here). However, without a no-acupuncture treatment group in the current study, we cannot rule out the natural reduction of the area of mechanical hyperalgesia over the period of the experiment. A no-treatment group will be needed in future studies.

Electroacupuncture in the current study was applied to the four extremities. At least one pair of electrodes cross the area of mechanical hyperalgesia. It is worth noting that there are many forms of acupuncture, such as auricular acupuncture and scale acupuncture. It is unknown if those forms of acupuncture that are far away from the hyperalgesia area have a different effect from the form used in the current study. Indeed, a previous animal study showed that REA on the hind paws was more effective in reducing mechanical hyperalgesia on the hind paw than REA on the hind leg or on the head (Kim et al., 2009). In addition, the magnitude of REA stimulation was adjusted individually to achieve to a strong but comfortable level with visible muscle contraction; but the actual intensity of REA stimulation in milliamp was not recorded. To enhance the reproducibility, it is necessary to record this parameter (Zheng et al., 2010). Interpretation of the results are limited to those constrains.

### Interpretation of Findings

The model used and the REA parameters used in this study were based on previous findings. We compared the results of current study with those studies. Drugs of various categories have been shown to have different effects on heat and mechanical hyperalgesia in the heat/capsaicin 45°C/0.075% model used in the current study. NMDA antagonists, that specifically target central sensitization reduced mechanical hyperalgesia, but had no effect on heat hyperalgesia (Duedahl et al., 2005; Mathiesen et al., 2006), whereas opioid medications that potentially impact on peripheral sensitization as well as central sensitization reduced both heat and mechanical hyperalgesia (Petersen et al., 2001, 2003). It is surprising that acupuncture, which analgesia has always been thought to mediate via the endogenous opioid system, had a positive effect on heat hyperalgesia, but not on mechanical hyperalgesia in this study.

Hyperalgesia model was successfully produced in the current study as indicated with a large area of mechanical hyperalgesia and an increased rating to nearly painful heat stimulation. HPT did not, however, reduce significantly after the application of capsaicin. This is largely due to the lower HPT on the application side prior to the hyperalgesic state. The HPT was 42.6 and 42°C prior to capsaicin for REA and SEA groups, respectively, and reduced slightly to 42.5 and 41.5°C afterward capsaicin. In the previous studies, the baseline HPT was 43–44°C then reduced to 42.8°C (Dirks et al., 2003). It is not clear why this happened as the HPT on the non-capsaicin side (43–44°C) in the current study was comparable to other studies.

Nevertheless, the hyperalgesia state produced in this study was comparable to those in the literature (Petersen-Felix et al., 1995; Petersen et al., 2001). In this model, mechanical hyperalgesia was thought to be maintained by heat stimulation, that is peripheral sensitization. The rekindling with heat produces sufficient C-nociceptor input to partially counteracts the natural reduction of mechanical hyperalgesia, thus achieving a long-lasting hyperalgesic state (Petersen and Rowbotham, 1999). Reducing heat hyperalgesia by cooling the capsaicin area reduces mechanical hyperalgesia (LaMotte et al., 1991; Koltzenburg et al., 1992). Consistent with the previous findings of this model, the magnitude of mechanical hyperalgesia in the current study was reduced as heat hyperalgesia was decreased. Although the reduction of heat hyperalgesia was more pronounced in REA group than SEA group, this difference did not lead to a more rapid reduction of mechanical hyperalgesia in the REA group. It is likely that the sustainment of mechanical hyperalgesia does not require a strong peripheral sensitization. Indeed, the relationship between heat and mechanical hyperalgesia in heat/capsaicin 45°C/0.075% model is not linear. A large area of mechanical hyperalgesia is often produced in this model whereas the reduction of HPT, indicating heat hyperalgesia, is small (Petersen et al., 2001, 2003). In addition, in older adults, the duration of mechanical hyperalgesia induced by topical application of capsaicin outlasted that of heat hyperalgesia. Those results support the notion that there is a lack of strong correlations between heat and mechanical hyperalgesia in capsaicin models.

The average VAS rating to thermal stimulation was approximate 6.5/10 on a VAS immediately after the establishment of the model in both groups. This rating is relatively low in a modified 0–10 VAS scale, 5 is defined as just painful and 10 as worst pain possible. It is unknown if the difference between REA and SEA will be larger if a model with a more severe pain and hyperalgesia is used. Previous studies show that acupuncture is more effective when pain is moderate to severe (Witt et al., 2011). It is well known that topical application of capsaicin with a higher concentration induces more pain than that with a lower concentration (Zheng et al., 2000). It is necessary to replicate the study in a heat/capsaicin model with a different combination of concentration of capsaicin and thermal stimulation so that the pain ratings to heat stimulation can reach 7 or 8 out of 10.

The results of this study are contradictory to the finding in the animal studies, in which acupuncture always outperformed sham acupuncture and furthermore, the non-invasive sham procedure consistently showed no anti-hyperalgesia effect (Lao et al., 2004). This discrepancy may be due to several factors. Firstly, the model in the animal study is often an inflammation model produced with an injection of complete Freund’s adjuvant (CFA); whereas the current heat/capsaicin 45°C/0.075% model is a neural model where both capsaicin and heat stimulations directly stimulating C fibers. Secondly, the magnitude of hyperalgesia in animal model is much larger compared with the human model. It is possible that at a high level of severity, the sham procedure is unable to produce any significant non-specific effect. Thirdly, the intensity of REA stimulation in the animal studies seems much stronger than that used in the studies in humans. It is clear from the animal studies that the higher the intensity, the stronger the effect of REA (Lao et al., 2004). Lastly, level of expectancy may also play a significant role as it has been found to contribute positively to the clinical outcome in patients with low back pain treated with acupuncture (Kalauokalani et al., 2001; Linde et al., 2007). It is unlikely that animals have any expectancy from the treatment, thus do not develop any expectancy-related placebo effect. However, the level of expectancy was not assessed in the current study. Indeed, SEA was well designed in the current study, that the participants could not tell if they received real or sham intervention. In addition, about 50% of those in SEA group reported mild to moderate pain. It is unclear if the pain induced by SEA has any physiological effects on inhibiting mechanical hyperalgesia, however, this expectation cannot be ruled out.

Our results also seem to be contradictory to a human study in which injection of capsaicin was used to assess acupuncture analgesia (Rebhorn et al., 2012). Rebhorn’s study found no difference between acupuncture and non-invasive sham acupuncture in reducing pain, flare, allodynia or mechanical hyperalgesia induced by capsaicin. The study differs from the current study in the form of acupuncture used, and the model and the type of outcome measures. We used EA in the current study, but Rebhorn et al. (2012) used manual acupuncture. Our previous study found that EA was far better than manual acupuncture in reducing experimental pain (Zheng et al., 2010). In the Rebhorn’s study, experimental pain model was induced during acupuncture treatment. The intensity of capsaicin injection-induced pain was strong and nearly 100 on 0–100 VAS rated by the participants. It is likely this strong pain interferes with the inhibitory effect of acupuncture. The intensity of pain also reduced rapidly within 10 min, giving insufficient time for acupuncture to show its effect. In the current study, EA was delivered after the model has been established; and the heat/capsaicin 45°C/0.075% model produces stable mechanical hyperalgesia that is long lasting and enable use to assess the anti-hyperalgesic effect of EA. Rebhorn’s study examined whether acupuncture could preempt a strong spontaneous pain, whereas in the current study we were interested in if EA could reduce hyperalgesia. In a capsaicin model, spontaneous pain is due to spontaneous firing of first order neurons where mechanical hyperalgesia is due to central sensitization (LaMotte et al., 1992). In summary, although Rebhorn’s and current studies share some similarities, they cannot be compared due to significant differences in the study design and the aims.

### Differential Effects of REA

The current study indicates that the effect of acupuncture could be dependent on the types of pain, in this case pain driven by peripheral or central sensitization. It is well understood that chronic pain, such as chronic low back pain, chronic headache and fibromyalgia, has a dominate central component, and may be not required to be peripherally driven (Woolf, 2011). Drugs that may modify central sensitization, such as anti-depressants, are effective for fibromyalgia (Perrot et al., 2008). This may explain why the effect between real and sham acupuncture is often very small in trials examining chronic pain. A meta-analysis of 17, 922 patients individual data has shown that the difference between real and sham acupuncture for chronic pain was 0.2 SD, reflecting a small effect (Vickers et al., 2012). In contrast, the effect of acupuncture on acute pain, such as post-operative pain, is often superior to sham acupuncture with an effect size about 0.7 SD (Cho et al., 2014; Wu et al., 2016). It is more like that postoperative pain is predominately driven by peripheral sensitization. Our study may explain this discrepancy.

We observed the effect of REA lasted for 1.5 h after the termination of acupuncture. The immediate effect of acupuncture was the best among all time points. After that, the rating to heat stimulation was slowly returning to pre-treatment hyperalgesia state in a similar manner in both EA and SEA, reflecting the effect of either intervention was of short-term. This seems to be contradictory to our previous study where the anti-temporal summation effect of EA became more evident post 24 h (Zheng et al., 2010). The current study only lasted for 4 h, thus does not allow sufficient time to assess the time profile of acupuncture.

### Implication for Future Studies

The potential disparity of acupuncture effects on mechanical and heat hyperalgesia indicates that this non-drug therapy may have a different impact on peripheral and central sensitizations. This may explain the varied findings in acupuncture trials for chronic and acute pains. The findings of this study are warranted to be further tested in other hyperalgesia models and in a larger sample. It will be ideal if the magnitude of the heat/capsaicin 45°C/0.075% model could be intensified with a higher concentration of capsaicin, and with a hyperalgesia state can last for more than 24 h, such as in a burn injury (Pedersen et al., 1996). Furthermore, participants’ expectancy for acupuncture should also be assessed in healthy human studies to determine its impact. Future acupuncture clinical trials should aim to assess peripheral and central sensitization components of the types of pain studied.

## Conclusion

We have shown that peripheral and central sensitizations in the heat/capsaicin 45°C/0.075% model responded to EA differently in healthy subjects, suggesting acupuncture analgesia could vary depending on the types of pain. This observation may explain some inconsistent findings from clinical trials of acupuncture and should be taken into consideration in the design of future acupuncture trials.

## Data Availability Statement

The datasets generated for this study are available on request to the corresponding author.

## Ethics Statement

This study involved human participants was reviewed and approved by the Human Research Ethics Committee of RMIT University (Reference No. 13/07). The patients/participants provided their written informed consent to participate in this study.

## Author Contributions

ZZ, LB, CL, and CX initiated the research project and developed the research protocol. ZZ, LB, and MO’L developed the acupuncture treatment protocol. LB conducted the experiment and all the outcome tests. MO’L delivered the acupuncture treatment. ZZ analyzed the data. ZZ and LB drafted the manuscript. All authors have commented on the drafts and approved the final version.

## Conflict of Interest

LB and MO’L conducted the research prior to being employed by company Spring Health. The remaining authors declare that the research was conducted in the absence of any commercial or financial relationships that could be construed as a potential conflict of interest.
